# Genetic variation of the Toll-like receptors in a Swedish allergic rhinitis case population

**DOI:** 10.1186/s12881-017-0379-6

**Published:** 2017-02-23

**Authors:** V. Henmyr, D. Carlberg, E. Manderstedt, C. Lind-Halldén, T. Säll, L. O. Cardell, C. Halldén

**Affiliations:** 1 Department of Biology, Lund University, Lund, Sweden; 2 Department of Biomedicine, Kristianstad University, 291 39 Kristianstad, Sweden; 3 Division of ENT Diseases, Department of Clinical Science, Intervention and Technology, Karolinska Institutet, Stockholm, Sweden

**Keywords:** Allergic rhinitis, Mutation spectrum, Next-generation sequencing, Rare variants, Toll-like receptor

## Abstract

**Background:**

Variation in the 10 toll-like receptor (TLR) genes has been significantly associated with allergic rhinitis (AR) in several candidate gene studies and three large genome-wide association studies. These have all investigated common variants, but no investigations for rare variants (MAF ≤ 1%) have been made in AR. The present study aims to describe the genetic variation of the promoter and coding sequences of the 10 TLR genes in 288 AR patients.

**Methods:**

Sanger sequencing and Ion Torrent next-generation sequencing was used to identify polymorphisms in a Swedish AR population and these were subsequently compared and evaluated using 1000Genomes and Exome Aggregation Consortium (ExAC) data.

**Results:**

The overall level of genetic variation was clearly different among the 10 TLR genes. The *TLR10*-*TLR1*-*TLR6* locus was the most variable, while the *TLR7*-*TLR8* locus was consistently showing a much lower level of variation. The AR patients had a total of 37 promoter polymorphisms with 14 rare (MAF ≤ 1%) and 14 AR-specific polymorphisms. These numbers were highly similar when comparing the AR and the European part of the 1000Genomes populations, with the exception of *TLR10* where a significant (*P* = 0.00009) accumulation of polymorphisms were identified. The coding sequences had a total of 119 polymorphisms, 68 were rare and 43 were not present in the European part of the 1000Genomes population. Comparing the numbers of rare and AR-specific SNPs in the patients with the European part of the 1000Genomes population it was seen that the numbers were quite similar both for individual genes and for the sum of all 10 genes. However, *TLR1*, *TLR5*, *TLR7* and *TLR9* showed a significant excess of rare variants in the AR population when compared to the non-Finnish European part of ExAC. In particular the *TLR1* S324* nonsense mutation was clearly overrepresented in the AR population.

**Conclusions:**

Most TLR genes showed a similar level of variation between AR patients and public databases, but a significant excess of rare variants in AR patients were detected in *TLR1*, *TLR5*, *TLR7*, *TLR9* and *TLR10*. This further emphasizes the frequently reproduced *TLR10*-*TLR1*-*TLR6* locus as being involved in the pathogenesis of allergic rhinitis.

**Electronic supplementary material:**

The online version of this article (doi:10.1186/s12881-017-0379-6) contains supplementary material, which is available to authorized users.

## Background

Allergic rhinitis (AR) is a global illness with a well-recognized impact on quality of life and work performance. It is characterized by nasal obstruction, secretion and itching and is often associated with symptoms of the eyes, fatigue and asthma [1]. The heritability of AR has been estimated to 0.66–0.78 [2, 3] and its development is generally believed to be the result of an interaction between genetical and environmental factors. Toll-like receptors (TLRs) are a well-established group of pattern-recognition receptors encompassing ten members which initiate innate immune responses [4]. Contrary to this, TLR10 has recently been shown to have inhibitory effects by down-regulating TLR2 mediated immune response [5]. The TLRs are an important barrier between environment and the organism and have been suggested to play a key role in the development of allergic disease in growing infants [6].

The 10 TLR genes are located on five different chromosomes. *TLR1*, *TLR6* and *TLR10* are located in a 90 kbp region on chromosome 4, *TLR7* and *TLR8* are located in a 57 kbp region on the X chromosome, whereas the remaining genes are located in separate loci on chromosome 1 (*TLR5*), chromosome 3 (*TLR9*), chromosome 4 (*TLR2* and *TLR3*) and chromosome 9 (*TLR4*). Earlier studies of genetic variation have reported a large number of polymorphisms for all 10 TLR genes [7, 8]. More recently, the 1000Genomes project has re-sequenced 1092 healthy individuals from 14 populations using low-coverage whole-genome and high-coverage exome sequencing. In concordance with previous studies, the 1000Genomes populations also show high levels of variation in the TLR genes [9].

The TLR genes have been significantly associated with various allergic phenotypes in linkage and candidate gene studies [10–14]. Single nucleotide polymorphisms (SNP) in *TLR7* and *TLR8* have been found to be associated with AR in two independent studies [15, 16]. Three meta-genome-wide association studies (GWAS) have analyzed various allergic phenotypes including self-reported AR and allergic sensitization [17–19]. A total of 37 loci were associated with allergic disease and four of these loci were reported by all three studies. The *TLR10-TLR1*-*TLR6* locus was one of these 4 loci. Following these studies, a replication attempt of the SNPs identified in the three meta-GWAS was performed using both the original phenotype definitions and a more strict AR definition [20]. A total of eight loci were successfully replicated. The *TLR10-TLR1*-*TLR6* locus was replicated using three index SNPs and all phenotype definitions, including a more strictly defined AR phenotype. Also, an additional meta-GWAS that analyzed a combined phenotype of asthma and hay fever identified a total of 10 loci, including the *TLR10-TLR1*-*TLR6* locus, as associated with the disease [21].

## Methods

The present study aims to describe the genetic variation of the promoter and coding sequences of the 10 TLR genes in a Swedish AR population of 288 patients. As the first larger resequencing effort in AR, this could identify rare variants that are increasing risk for AR and identify potential targets for future studies.

### Study samples

The 288 AR patients (140 females, 148 males, mean age 33 years) were recruited at Malmö University Hospital (Malmö, Sweden) between the years 2003 and 2009 and consist of unrelated individuals from the general population. All patients were of Caucasian origin, with both parents born in Sweden. They were patients at the allergy clinic and were diagnosed with symptomatic birch and/or timothy grass pollen induced intermittent AR (for more details see Additional file 1a). As described in Nilsson et al. 2012, diagnostic procedures for the study population included face-to-face personal interview of medical history and skin prick tests (SPT) [22] or Phadiatop tests with at least a class two response to birch and/or timothy grass pollen. A total of 59% of the patients showed a positive SPT for both allergens. SPT were performed with a standard panel of 11 common airborne allergens (ALK-Abelló, Hörsholm, Denmark) (for more details see Additional file 1b). SPT were performed on the volar side of the forearm with saline buffer as negative and histamine chloride (10 mg/ml) as positive controls. A wheal reaction diameter of ≥3 mm was considered a positive SPT response. Atopy is defined as a positive SPT reaction to at least one of the tested allergens and AR is diagnosed based on the presence of atopic status and typical AR symptoms as defined by the Allergic Rhinitis Impact on Asthma (ARIA) guidelines. None of the patients suffered from severe asthma and less than 10% had moderate asthma with continuous medication. Genomic DNA was isolated from blood collected in EDTA using the QIAmp DNA Blood kit (Qiagen, Hilden, Germany) and DNA concentrations were determined by fluorometry using PicoGreen (Molecular Probes, Eugene, OR, USA).

### Sanger sequencing

Primers were designed using NCBI Primer-BLAST (http://www.ncbi.nlm.nih.gov/tools/primer-blast/) to amplify at least 50 bp downstream and 500 bp upstream of the start of exon 1 of all 10 TLR genes (for exact coordinates and primer sequences see Additional files 2 and 3). Big Dye Terminator Sanger sequencing was performed in both directions using a 3130XLGenetic Analyzer (Applied Biosystems, Foster City, CA, USA). Sequences were interpreted and all polymorphisms were identified using SeqScape ver. 2.5 (Applied Biosystems) and confirmed by manual inspection. Further information about Sanger sequencing can be found in Additional file 4.

### Ion Torrent sequencing

The primer sets used in this study were obtained from Ion AmpliSeq™ Designer (http://www.ampliseq.com, pipeline version 2.0.3). A total of 204 systems were designed covering 98.8% of the coding sequence of *TLR1-TLR10* (for exact coordinates and primer sequences see Additional files 2 and 5). Template DNA was pooled such that each pool contained equimolar amounts of DNA from 12 individuals, producing a total of 24 pools for the 288 AR patients. Next-generation sequencing (NGS) was performed using an AmpliSeq strategy on an Ion Torrent PGM platform (Life Technologies, Carlsbad, CA, USA). The sequences were aligned against the human reference sequence (build GRCh37) using Torrent Suite 3.6 and primer sequences were trimmed away. Variant calling was then performed using variant calling parameters tuned for high sensitivity. Annotation of the variant SNPs was accomplished by submitting them to SeattleSeq Annotation 137 (http://snp.gs.washington.edu/SeattleSeqAnnotation137/). Further information about Ion Torrent sequencing can be found in Additional file 4.

### Genetic analysis

Publically available information on the polymorphisms in the 10 TLR genes were extracted from dbSNP (http://www.ncbi.nlm.nih.gov/SNP/) and from the Integrated Variant Set of the 1000Genomes Project (http://ftp.1000genomes.ebi.ac.uk/vol1/ftp/release/20110521/) release April 2012 [date (09, 2014) accessed]. The 1000Genomes data set was then subdivided into four separate populations; individuals of European (EUR; 379 individuals), African (AFR; 246), Asian (ASN; 286) and South American origin (AMR; 181). Allele frequencies were calculated for each variation using the same allele as referent for all populations. Missense mutations identified in the study population and in the 1000Genomes population were investigated using SIFT [23] and PolyPhen-2 [24].

Three different statistics describing the spectrum of variation were calculated to investigate for the accumulation of rare TLR variants in AR patients. The first statistic calculated the number of sites where the minor allele frequencies (MAF) were ≤ 1% in patients (AR population) and controls (EUR population). The second statistic calculated the number of variants that were unique to either AR patients or EUR controls and the third statistic compared SNPs detected in patients and controls using information obtained in SIFT and PolyPhen-2 analysis. A one-sided permutation test was used to test all three statistics for equality of the populations and the three statistics were summarized for each individual gene and for the sum of all genes. One-sided tests were used since it was the possible accumulation of rare variants in the AR-population that was under investigation. The permutation test randomized the alleles among patients (AR population) and controls (EUR population) for each variable site and each of the three statistics were calculated 100 000 times. The *P*-values of the tests were equal to the proportions of simulations where the randomized AR population had a higher value than the value of the actual AR population. A more detailed description can be found in Additional file 4. Data from the Exome Aggregation Consortium (ExAC) (Cambridge, MA (URL: http://exac.broadinstitute.org) [date (03, 2015) accessed]) were also used to test for accumulation of rare variants in the coding regions of the ten TLR genes. The non-Finnish European population of ExAC consists of > 30.000 individuals and were used for the extraction of polymorphisms (excluding indels) present in the coding region of the ten TLRs. A one-sided simulation test was used to investigate for the probability of an excess of rare variants in the AR patients compared to ExAC data.

Polymorphisms where at least one of the AR and EUR populations showed a MAF ≥ 0.05 were also tested for association with AR using the normal approximation test. For detailed descriptions of bioinformatics and genetic analysis see Additional file 4, and for an overview of the study outline see Additional file 6.

## Results

### Screening for polymorphisms in the promoters of the 10 TLR genes

The 288 AR patients were screened for polymorphisms in the putative promoter regions of the 10 TLR genes using Sanger sequencing in both directions. The promoter regions were defined as the region 50 bp downstream to 500 bp upstream of the start of exon 1 of all the genes. The sequence data was generally of high quality with > 95% of bases having a Phred score of 30 or higher in > 95% of individuals. *TLR2* and *TLR5* had slightly lower quality scores with > 90% of bases having a Phred score of 20 or higher in > 75% of individuals. A total of 37 polymorphisms were detected, 25 of these were present in dbSNP and 12 were not (Table 1 and for complete table see Additional file 7). The 10 genes varied drastically with respect to the number of detected polymorphisms with *TLR10* harbouring the highest number of variants (13), equalling 35% of all detected polymorphisms. *TLR1* and *TLR6* which both reside in the same locus as *TLR10* also show higher than average (3.7) numbers of polymorphisms (5 polymorphisms each). *TLR7* and *TLR8* which are located in a small region on the X chromosome, both show lower numbers of polymorphisms (1 and 2 polymorphisms, respectively).

**Table 1:** Number of polymorphisms detected in the promotor regions of the *TLR1*-*TLR10* genes. Promoter region is for both populations defined as 50 bp downstream and 500 bp upstream of the start of exon 1. The AR population contains 288 individuals and the EUR population contains 379 individuals

	AR population			EUR population		
Gene	Total	≤1%^a^	AR-specific^b^	Total	≤ 1%^a^	EUR-specific^c^
*TLR1*	5	1	1	4	0	0
*TLR2*	3	1	0	6	3	3
*TLR3*	4	3	3	1	1	0
*TLR4*	0	0	0	2	2	2
*TLR5*	2	0	0	4	2	2
*TLR6*	5	1	1	9	4	5
*TLR7*	1	1	1	2	1	2
*TLR8*	2	0	0	3	1	1
*TLR9*	2	1	2	2	1	2
*TLR10*	13	6	6	7	0	0
Total	37	14	14	40	15	17

^a^Polymorphisms with minor allele frequencies ≤ 1%

^b^Polymorphisms present in the AR population and not present in the EUR population

^c^Polymorphisms present in the EUR population and not present in the AR population

The corresponding sequence data from 379 individuals with European ancestry (EUR population) were extracted from the 1000Genomes project and compared with data from the AR patients (Table 1 and for exact coordinates used see Additional file 2). The total number of polymorphisms per gene was similar in the two populations, with a correlation coefficient of 0.6. A major part of this similarity is made up of the 23 polymorphisms that are in common to the AR and EUR populations. These polymorphisms have in general high MAFs; 15 SNPs > 0.05, 7 SNPs 0.01–0.05 and 1 SNP < 0.01 (Additional file 7) and since both populations are samples from European populations this is not unexpected. None of the promoter SNPs with MAF ≥ 5% showed any significant deviation from Hardy-Weinberg equilibrium. When investigating these SNPs for allele frequency differences between the AR and EUR populations, only one polymorphism (rs3764879) in *TLR8* with an allele frequency difference of 0.09 yielded an uncorrected *P*-value < 0.05. The number of rare (MAF ≤ 1%) and population-specific variants do not show a positive correlation between the populations but they are on the other hand so few that the correlation coefficient is uninformative. Two things are directly apparent from Table 1, the total numbers of rare and population-specific variants are relatively low and the sums of the different categories are very similar when comparing the AR and the EUR populations. Thus, already at this level the hypothesis of a strong general accumulation of rare variants in the AR patients can be rejected. This is largely supported by the results from the permutation test (Table 2). In *TLR10* there is both a higher number of SNPs with MAFs ≤ 1% and a higher number of population-specific SNPs in AR patients. This yields a *P*-value of 0.00009 for AR-specific SNPs that is sufficiently low to pass a Bonferroni correction (Table 2). It should be noted that since the two categories are to a large extent overlapping, the tests are highly dependent. Thus, a Bonferroni correction based on 20 tests would be very conservative. In addition, the test of the total number of AR-specific SNPs yields an uncorrected *P*-value of 0.03 in *TLR3*.

**Table 2:** Probability (Psim) for accumulation of polymorphisms in AR population relative to EUR population

Gene	*P*_sim_ (≤ 1%)	*P*_sim_ (AR-specific)
*TLR1*	0.42	0.42
*TLR2*	0.99	1.00
*TLR3*	0.28	0.03
*TLR4*	1.00	1.00
*TLR5*	1.00	1.00
*TLR6*	1.00	0.82
*TLR7*	0.98	0.19
*TLR8*	1.00	1.00
*TLR9*	0.90	0.18
*TLR10*	0.02	0.00009
Sum of all SNPs	0.94	0.004

### Screening for polymorphisms in the coding sequences of the 10 TLR genes

The Ion Torrent sequencing of the 288 AR patients identified a total of 119 polymorphisms in the coding sequences of the 10 TLR genes, 68 with an allele frequency ≤ 1%, 43 not present in the EUR population and 21 not previously described in dbSNP (Table 3 and see Additional file 8 for a complete list of variants). Similarly to what was found for the promoter sequences, the coding sequences differ strongly with respect to the number of polymorphisms; also in this case *TLR10* shows the highest number (18) with *TLR1* (14) and *TLR6* (11) again showing high numbers of polymorphisms. Also in this case *TLR7* (8) and *TLR8* (9) showed a lower than average (11.9) number of variable sites.

**Table 3:** Number of polymorphisms detected in the coding regions of *TLR1*-*TLR10*. The AR population contains 288 individuals and the EUR population contains 379 individuals

		AR population								EUR population						
Gene	Base-pairs	Total	≤ 1%^a^	AR-specific^b^	New^c^	Missense	Nonsense	Synonymous	Damaging^d^	Total	≤ 1%^a^	EUR-specific^e^	Missense	Nonsense	Synonymous	Damaging^d^
*TLR1*	2358	14	8	4	1	9	1	4	3	19	13	9	14	0	5	6
*TLR2*	2352	10	5	4	1	6	1	3	3	10	5	4	4	0	6	2
*TLR3*	2712	8	6	4	4	6	0	2	4	12	10	8	7	0	5	4
*TLR4*	2517	11	9	5	2	5	0	6	2	13	11	7	9	0	4	2
*TLR5*	2574	15	9	4	1	10	1	4	1	17	11	6	11	2	4	2
*TLR6*	2388	11	6	5	3	7	0	4	1	16	11	10	12	0	4	3
*TLR7*	3147	8	5	4	1	5	0	3	1	5	2	1	3	0	2	1
*TLR8*	3123	9	3	2	2	2	0	7	0	9	2	2	1	0	8	0
*TLR9*	3096	15	13	9	5	6	0	9	3	15	13	9	6	1	8	2
*TLR10*	2433	18	4	2	1	13	0	5	3	26	12	10	16	1	9	4
Total	2358	119	68	43	21	69	3	47	21	142	90	66	83	4	55	26

^a^Polymorphisms with minor allele frequencies ≤ 1%

^b^Polymorphisms present in the AR population and not present in the EUR population

^c^Polymorphisms not present in dbSNP

^d^Concordant classification according to SIFT and PolyPhen-2

^e^Polymorphisms present in the EUR population and not present in the AR population

A comparison of the results of the AR and EUR populations shows that both the total number of polymorphisms and the number of rare polymorphisms are slightly higher in the EUR population, 119 versus 142 and 68 versus 90 (Table 3). The distribution of the individual polymorphisms over the TLR genes is very similar between the AR and EUR populations, with a correlation coefficient of 0.89. Looking at the rare SNPs there is still a positive correlation between the two populations. Comparing the numbers of rare and AR-specific SNPs it is seen that the numbers are quite similar both for individual genes and for the sum of all 10 genes. When a permutation test was applied to test for accumulation of variants in the AR population, only the test for AR-specific polymorphisms in *TLR7* was significant with an uncorrected *P*-value of 0.02 (Table 4). Also the overall test of the AR-specific polymorphisms gave a significant result (*P* = 0.04) out of a total of 22 tests performed.

**Table 4:** Probability (Psim) for accumulation of polymorphisms in AR population relative to EUR population and the non-Finnish European population of ExAC

Gene	*P*_sim_ (≤ 1%)	*P*_sim_ (AR-specific)	*P*_sim_ (ExAC ≤ 1%)
*TLR1*	0.91	0.63	0.012
*TLR2*	0.51	0.25	0.62
*TLR3*	0.76	0.79	0.32
*TLR4*	0.80	0.24	0.37
*TLR5*	0.71	0.18	0.042
*TLR6*	0.97	0.56	0.75
*TLR7*	0.25	0.020	0.0034
*TLR8*	0.68	0.14	0.91
*TLR9*	0.53	0.16	0.00086
*TLR10*	0.91	0.91	1.00
Sum of all SNPs	0.91	0.04	0.42

The non- Finnish European population of ExAC were used as an additional control population. Due to the different structure of the data it was used in a simulation test to investigate the probability for an accumulation of rare variants (MAF ≤ 1%) in the AR population. As in the test for AR-specific variants vs the EUR population, *TLR7* again shows a significant result and yields an uncorrected *P*-value of 0.0034. In addition, *TLR1*, *TLR5* and *TLR9* also gave rise to significant results with *TLR9* showing the lowest *P*-value, indicating an excess of rare variants in the AR population compared to ExAC data.

The analysis above covered all variants. An obvious alternative is to focus on variants that alter the gene products i.e. 3 nonsense mutations and 69 missense mutations. Forty-four out of the 69 missense mutations had MAFs ≤ 0.01. *TLR1*, *TLR5* and *TLR10* had the highest numbers of non-synonymous variants with 10, 11 and 13 variants, respectively. The 3 nonsense mutations were S324* in *TLR1*, R447* in *TLR2* and R392* in *TLR5* (Additional file 8). The nonsense mutation S324* in *TLR1* had an allele frequency of 0.009 in the AR population (estimated to represent 5 copies), whereas it was not present at all in the EUR population or the non-Finnish population of ExAC (investigating 66 616 chromosomes). The corresponding frequencies for the R447* mutation in *TLR2* was estimated to a single copy in the AR population and none in the EUR population. In *TLR5* the estimated allele frequencies of the R392* mutation were similar at 0.059 and 0.060 in the AR and EUR populations, respectively. In order to evaluate a potential accumulation of damaging variants, a permutation test based on the SIFT and PolyPhen-2 scores were used. The one-sided test showed no significant differences between the AR and EUR populations.

SNPs with MAF ≥ 5 were also tested individually for allele frequency differences between the AR and EUR populations. Two SNPs showed significant allele frequency differences and were located in *TLR5* (rs2072493) and in *TLR8* (rs3764880), respectively. Both were missense mutations classified as “benign” by PolyPhen-2. The allele frequency differences were 0.056 and 0.086 corresponding to *P*-values of 0.04 and 0.008, respectively. In both cases, the AR population had a lower frequency of the rare allele.

## Discussion

**Fig. 1:**
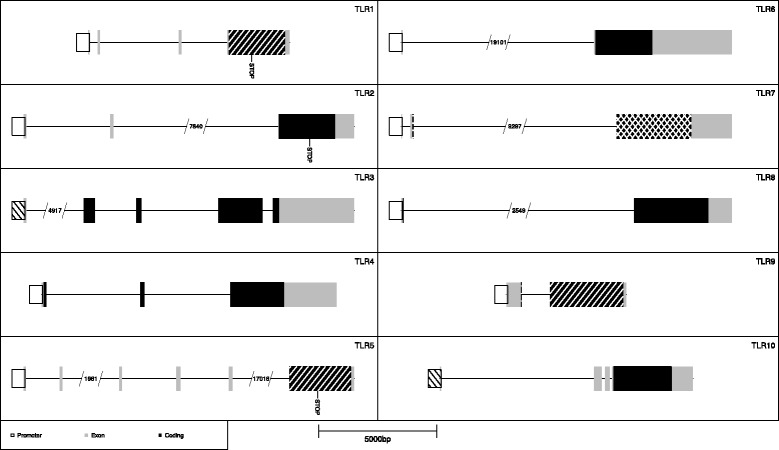
Results summary for the TLR genes. Significant differences between AR patients and 1000Genomes data are indicated with diagonal lines going from top left to bottom right. Significant differences between AR patients and ExAC data are indicated with diagonal lines going from top right to bottom left. Nonsense mutations are denoted with “STOP”. Truncated introns are indicated with gaps and the corresponding sequence lengths are given

We have adopted a broad perspective in the present study where we investigate all 10 TLR genes for both rare (MAF ≤ 1%) and common (MAF > 1%) polymorphisms in both promoters and coding sequences. The polymorphisms are also subdivided into different classes e.g. nonsense and missense where missense mutations are further evaluated based on functional predictions. The primary objective of the present study was to describe the genetic variation of the TLR genes in a Swedish AR population in comparison to the general variation seen in background populations similar to our own. A second objective was to identify rare candidate mutations that are increasing risk for AR. The main results of this study is summarized in Fig. 1.

In the present study different sequencing methods were used to create the different datasets. Since different sequencing platforms have different error profiles, direct comparisons can be problematic. The TLR exome data analyzed in this study have high coverage (>50X) in 1000Genomes and ExAC data, and in our Ion Torrent sequencing data (~200X), for more information see [Additional file 9]. In addition, we have chosen not to report any indels for the TLR exome as Ion Torrent sequencing is notorious for its high error rate with respect to indels. Thus, the probability of errors is expected to be low in the different datasets. Sanger sequencing of the promoter regions generally yielded high quality data and the identified polymorphisms are likely to be true. Two exceptions exist, both *TLR2* and *TLR5* show slightly lower quality, but since these two genes do not give rise to any significant signals these genes were not evaluated further. The low coverage (2–6X) NGS data from the 1000Genomes project are more likely to create both false positive and false negative data. However, since both the total number and the number of rare polymorphism are highly similar for the two populations, this seems not to be a large problem. Another concern is the use of publicly available background populations as control groups since AR is a common disease with a prevalence of 10–20% [1]. However, the presence of AR cases among the controls only lower the power of the analysis. This is true, both for analysis of allele frequencies of common variants as well as the analysis of rare variants. Another concern related to this is the issue of population specific variants with increased allele frequencies. But since comparisons and tests are made using the collective number of alternative alleles from all rare variants in a specific region, and not the frequencies of single variants, the effect of population specific variants will be less pronounced.

A general observation that is not directly related to the occurrence of AR is the fact that the overall level of genetic variation is clearly different among the 10 TLR genes and that this to a large extent is related to their chromosomal locations. The *TLR10* gene is the most variable in both populations, in the promoter as well as in coding regions. The other two genes in the same region, *TLR1* and *TLR6*, are also well above average with regard to the number of variable sites. The other extreme is represented by *TLR7* and *TLR8* which are consistently showing a lower level of variation. These differences may be due to several different factors. It should be remembered that even under strict neutrality the level of variation is expected to vary between regions, i.e. the level of variation observed may be due to chance events. X-linked loci have slightly lower effective population size which may have contributed to the lower level of variation in *TLR7* and *TLR8*. In addition, since active genes are involved it can safely be assumed that purifying selection is operating on nonsense and some of the missense mutations. If the effects of these changes are recessive, the effect of selection is stronger on X-linked loci, thus further reducing the level of variation. This effect will be present for all sites in linkage disequilibrium with the selected loci.

Only a handful of the more frequent polymorphisms (MAF ≥ 5%) that could be tested individually yielded significant differences between the two investigated populations. SNPs in the promoter and the coding sequence of *TLR8* showed significant frequency differences between the AR and the EUR population. These SNPs are all located in the same haploblock and reflects the association observed earlier for the *TLR7*-*TLR8* region [16]. However, no signs of any accumulation of rare variants was detected in *TLR8* which was also demonstrated in a previous study [25].

Many of the missense variants in the coding sequence may well be damaging and result in lowered fitness. However, it is likely that a majority of these are not involved in the development of AR since approximately equal numbers were found in cases and controls. The 3 nonsense polymorphisms (*TLR1* S324*, *TLR2* R447* and *TLR5* R392*) are located in the leucine-rich repeats and truncates the receptors so that neither the transmembrane nor the toll-IL1 receptor domain will be translated and are therefore damaging to the function of the proteins. The *TLR1* S324* nonsense mutation was clearly overrepresented in the AR population compared to the EUR population and the non-Finish European ExAC population. The frequency observed in AR patients for S324* is 45 times higher than the most common nonsense mutation in *TLR1* in the complete ExAC database. Thus, the *TLR1* S324* mutation is a strong candidate for being associated with AR.

Investigation of rare variants in the promoter regions showed no general accumulation in AR patients relative to EUR controls. The major exception was the promoter region of *TLR10* where a surplus of 6 polymorphisms were identified in AR patients compared to controls. This difference was highly significant. In addition, SNPs in this region have previously been associated with altered expression of the *TLR10* gene [26], suggesting that altered gene expression of *TLR10* may be involved in the pathogenesis of allergic disease. Recently it was shown that TLR10 is a receptor with suppressive effects, reducing TLR2-mediated cytokine production including IL-1β, IL-6, IL-8 and TNF-α. This is believed to be achieved through either or all of the 3 following mechanisms; 1) competitive binding of ligands of stimulatory TLRs 2) TLR10 competing for hetero-dimerization of other TLR2 family members (TLR1 and TLR6) 3) direct inhibitory effects of TLR10 via P13k signaling pathway [5].

The analysis of the coding sequence showed no strong general accumulation of rare or AR-specific polymorphisms in AR patients relative to EUR controls. However, in the simulation tests for rare variants using ExAC data, *TLR1*, *TLR5*, *TLR7* and *TLR9* showed significant accumulations of rare variants in AR patients. SNPs in the *TLR1* locus have previously been associated with allergic disease in a number of large meta-GWAS [17–19, 21] and in a Swedish replication study [20]. Genetic variation in *TLR7* have been associated with the development of asthma, rhinitis, and AD in a Danish population [15] and also with skin-prick test response for house dust mites in a Singaporean Chinese population [16]. In a study of Tunisian children, SNPs in *TLR9* were reported to play a role in the predisposition to asthma [14] and were also associated with asthma risk in a recent meta-study [27]. Thus, the results from the simulation tests in the present study identifies genes previously shown to be associated with allergic diseases, but further studies investigating genetic variation in these genes and its contribution to allergic diseases are needed.

Three large meta-GWAS identified a total of 37 loci associated with AR where four of these loci were reported by all three studies [17–19]. One of these four loci was the *TLR10*-*TLR1*-*TLR6* locus which was identified by a large number of SNPs. Following these studies, a replication attempt of the SNPs identified in the three meta-GWAS was performed using both the original phenotype definitions and a more strict AR definition [20]. A total of eight loci were successfully replicated including the 90 kbp *TLR10*-*TLR1*-*TLR6* locus. This locus produced significant *P*-values for SNPs located in a region that were larger than 100 kbp and encompassed all three genes. Another meta-GWAS investigating a combined phenotype of asthma and hay fever also identified this locus as associated with the disease [21]. In addition, SNPs in this region have earlier been associated with altered expression levels of these genes [26]. The patterns of eQTL data for *TLR10*, *TLR1* and *TLR6* expression are shown in Additional file 10 together with the AR-associated SNPs from the meta-GWAS of Hinds et al. [19]. If the pattern of these signals are compared with the corresponding pattern from the AR association study of Hinds et al. [19] there is a striking similarity, indicating that the expression differences in the *TLR10*-TLR1*-TLR6* region is correlated with AR. The overrepresentation in the AR population of a set of variants in the promoter of *TLR10* and in the coding region of *TLR1* including the S324* mutation, identify these polymorphisms as candidate mutations and add further details to the associations observed for common SNP from this region in the meta-GWAS and the replication study. The lack of significant associations of common variants in the *TLR10-TLR1-TLR6* locus is probably a matter of power in the present study. There are in fact allele frequency differences for a number of SNPs, but at a non-significant level. Nonetheless, the rare variants found in this study coupled with previous strong association to common variants highly implicate the *TLR10-TLR1-TLR6* region as a risk-locus for AR.

## Conclusions

This study was motivated by the lack of re-sequencing studies in allergic diseases. Previous association studies have identified common variants in the TLR genes to be associated with AR. The present study describes the genetic variation of these genes in a population selected for AR, which is an important step to understand the genetic contribution of the disease. Also, the present study identifies an excess of rare variants in the *TLR1*, *TLR5*, *TLR7*, *TLR9* and *TLR10* genes in AR patients compared to public background populations, indicating that rare variants may also contribute to the disease. Further studies investigating the contribution of rare variants in AR using larger populations consisting of matched cases and controls are much needed, but the results of the present study may serve as a starting point for future studies.

## Additional files

Additional file 1: Demographics of study populations. (XLSX 9 kb)
Additional file 2: Targeted genomic regions. (XLSX 14 kb)
Additional file 3: Sanger sequencing primers. (XLSX 9 kb)
Additional file 4: Materials and methods for Sanger sequencing, Ion Torrent sequencing, Bioinformatics analysis and Genetic analysis. (DOCX 20 kb)
Additional file 5: Ion Torrent sequencing primers. (XLSX 22 kb)
Additional file 6: Outline of the study. (DOCX 12 kb)
Additional file 7: Characteristics of promoter polymorphisms detected in 288 AR patients and comparison with allele frequencies in subpopulations extracted from 1000Genomes project. (XLSX 22 kb)
Additional file 8: Characteristics of coding sequence polymorphisms detected in 288 AR patients and comparison with allele frequencies in subpopulations extracted from 1000Genomes project. (XLSX 67 kb)
Additional file 9: Ion Torrent coverage. (EPS 67 kb)
Additional file 10: eQTL and association data for *TLR10*-*TLR1*-*TLR6* locus. (EPS 228 kb)

## Abbreviations

AFR: African
AMR: South American
AR: Allergic rhinitis
ARIA: Allergic rhinitis impact on asthma
ASN: Asian
EUR: European
ExAC: Exome aggregation consortium
GWAS: Genome-wide association study
MAF: Minor allele frequency
NGS: Next-generation sequencing
SNP: Single nucleotide polymorphism
SPT: Skin prick test
TLR: Toll-like receptor
