# Supplementary material for: Genetic variation of the Toll-like receptors in a Swedish allergic rhinitis case population
Source: BMC Med Genet. 2017 Feb 23;18:18. doi: 10.1186/s12881-017-0379-6 (PMC5322632; doi:10.1186/s12881-017-0379-6)
Supplement: Additional file 4: — Materials and methods for Sanger sequencing, Ion Torrent sequencing, Bioinformatics analysis and Genetic analysis. (DOCX 20 kb) [file 12881_2017_379_MOESM4_ESM.docx]

## Additional file 4

## Sanger sequencing

Primers were designed using NCBI Primer-BLAST (http://www.ncbi.nlm.nih.gov/tools/primer-blast/) and purchased from DNA Technology A/S (Risskov, Denmark). The primer systems were designed to amplify at least 50 bp downstream and 500 bp upstream of the start of exon 1 of *TLR1-TLR10* (Table S1). Primary PCR was performed using KAPA Taq Extra HS PCR Kit (KAPA Biosystems, Cape Town, South Africa) and the following protocol: 1 cycle of 94°C for 3 min, 35 cycles of 94°C for 15 s, incubation at the annealing temperature for 15 s and 72°C for 1 min, and then 1 cycle of extension at 72°C for 10 min with a final soak at 4°C. Big Dye Terminator Sanger sequencing was performed in both directions using a 3130XL Genetic Analyzer (Applied Biosystems, Foster City, CA, USA). Primary PCR products were treated with ExoSAP-IT® (Applied Biosystems) and DNA sequencing was subsequently performed in a total volume of 5 µl containing 0.5X Big Dye sequencing ready reaction premix (Big Dye Terminator v 2.0, Applied Biosystems), 0.5X Big Dye Sequencing buffer and 3.2 pmol of the sequencing primer. The following PCR conditions were used: 1 cycle of 96°C for 1 min, 25 cycles of 96°C for 10 s, 50°C for 5 s and 60°C for 4 min. The sequencing reactions were purified using Xterminator (Applied Biosystems) according to the manufacturer’s instructions. Sequences were interpreted and all polymorphisms were identified using SeqScape ver. 2.5 (Applied Biosystems) and confirmed by manual inspection.

## Ion Torrent sequencing

The primer sets used in this study were obtained from Ion AmpliSeq™ Designer (http://www.ampliseq.com, pipeline version 2.0.3). A total of 204 systems were designed covering 98.8% of the coding sequence of *TLR1-TLR10* (Table S2). DNA sequencing was performed using an AmpliSeq strategy on an Ion Torrent PGM platform (Life Technologies, Carlsbad, CA, USA). Template DNA was pooled such that each pool contained equimolar amounts of DNA from 12 individuals, producing a total of 24 pools for the 288 AR patients. Initial amplification of the targeted regions was performed using the Ion AmpliSeq™ Library Kit 2.0 in 10 µl PCR reactions using 20 ng of template DNA. The PCR amplifications were made according to the following PCR protocol: 1 cycle of 99^o^C for 2 min, 17 cycles of 99^o^C for 15 s and 60^o^C for 4 min. Adapters were subsequently ligated to each pool of amplicons and clean-up and size selection was performed using Agencourt Ampure XP beads (Beckman Coulter, Indianapolis, IN, USA). DNA concentration and fragment size distribution of each library was determined by capillary electrophoresis on a Fragment Analyzer (Advanced Analytical Technologies Inc, Ames, IA, USA). The libraries were then diluted to a concentration of 35 pM and emulsion PCR performed using the OneTouch 2 machine with the Ion PGM Template OT2 200 kit. Templated spheres were recovered using Ion PGM Enrichment Beads. The samples were centrifuged at 15 500 x *g* and 3 µl of the re-suspended pellets were mixed with 3 µl of sequencing primer from Ion PGM Sequencing 200 kit v2. The sequencing primer was annealed by incubation at 95^o^C for 2 min and 37^o^C for 2 min followed by the addition of 1 µl of sequencing polymerase. The samples were loaded onto an Ion PGM 314 chip v2 and sequencing performed on an Ion Torrent PGM using the default flow order. The sequences were aligned against the human reference sequence (build GRCh37) using Torrent Suite 3.6 and primer sequences were trimmed away. Variant calling was then performed using variant calling parameters tuned for high sensitivity. Annotation of the variant SNPs was accomplished by submitting them to SeattleSeq Annotation 137 (<http://snp.gs.washington.edu/SeattleSeqAnnotation137/>).

A total of 204 amplicons were used to cover 28.2 kbp (98.8%) of the coding sequence of the 10 TLR genes and each 314 sequencing chip analysed one pool of 12 individuals. The average number of reads per chip was > 500 000 and summed over all chips this produced in total 1.7 Gbp of DNA sequence with > 63% of the reads at AQ20 and an average uniformity of 97%. Assuming that all primer pairs had equally effective amplification, all amplicons are expected to generate the same number of reads. Figure S1 shows the mean number of total reads per individual for each amplicon. Only 5 out of the 204 amplicons did not reach 50X coverage per individual on average. The coding sequence of the *TLR8* gene was also determined in the 288 AR patients using Sanger sequencing and the resulting data were compared with the corresponding data from the Ion Torrent sequencing. A total of 9 SNPs were detected by both strategies and no false positives or negatives were detected in the comparison of the two data sets. The allele frequencies of the 9 different SNPs varied between 0.01 and 0.35 and the allele frequency estimates were highly similar for the two methods. This strongly indicates high quality in the Ion Torrent data.

**Bioinformatics**

Publically available information on the polymorphisms in the 10 TLR genes were extracted from dbSNP (http://www.ncbi.nlm.nih.gov/SNP/) and from the Integrated Variant Set of the 1000Genomes Project (http://ftp.1000genomes.ebi.ac.uk/vol1/ftp/release/20110521/) release April 2012 [date (09, 2014) accessed]. Variants of the promoters and coding regions of the 10 genes were extracted for 1092 individuals using tabix [1]. The average coverage of the sequence data was > 50X in the coding regions and 2-6X in the promoter regions [2]. Using VCFtools [3] this data set was then subdivided into four separate populations; individuals of European (EUR; 379 individuals), African (AFR; 246), Asian (ASN; 286) and South American origin (AMR; 181). The EUR population can be further subdivided into five populations: 89 individuals from England and Scotland (GBR), 85 from Utah with northern and western European ancestry (CEU), 98 from Italy (TSI), 93 from Finland (FIN) and 14 from Spain (IBS). Allele frequencies were calculated for each variation using the same allele as referent for all populations. Missense mutations identified in the study population and in the 1000Genomes population were investigated using SIFT [4] and PolyPhen-2 [5].

**Genetic analysis**

Three different statistics describing the spectrum of variation were calculated to investigate for the accumulation of rare TLR variants in AR patients. The first statistic calculated the number of sites where the minor allele frequencies (MAF) were ≤ 1% in patients (AR population) and controls (EUR population). This statistic tests for accumulation of rare variants in general, where rare variants are defined as having MAF < 1%. The second statistic calculated the number of variants that were unique to either AR patients or EUR controls. This statistic tests for accumulation of disease specific variants of any frequency. The third statistic compared SNPs detected in patients and controls using information obtained in SIFT and PolyPhen-2 analysis. SIFT classify mutations into tolerated and damaging, which were given the scores 0 and 1, respectively. PolyPhen-2 classify mutations into benign, possibly damaging and probably damaging, which were given the scores 0, 1 and 2, respectively. The index was then simply the sum of these values for each site. This third statistic was constructed to detect accumulation of variants that impairs the function of the proteins. Together the three statistics tries to explain what variation there is and if it has any functional relevance. The first two statistics were applied to both promoter and coding sequences. The third statistic is of course only applicable to coding sequences. The three statistics were summarized for each individual gene and for the sum of all genes.

A permutation test was used to test all three statistics for equality of the populations. The alleles of the patients (AR population) and controls (EUR population) were pooled for each variable site. The alleles were then randomly assigned to the two populations. This was done for each of the three statistics and were calculated 100 000 times. The *P*-values of the tests were equal to the number of times where the randomized AR population had a higher value than the value of the actual AR population divided by the number of iterations (100 000). Thus, creating one-sided tests ignoring the possibility of higher values for the EUR population. For the promoter sequences in the AR population, sequence data was available at the level of the individual, whereas the allele frequencies for the coding sequences were estimated from pools of individuals. In the latter case, the estimates of the allele frequencies times the number of investigated individuals (rounded to the nearest integer) were used in the permutation tests. All data was available at the level of the individual in the EUR population.

Data from the Exome Aggregation Consortium (ExAC) (Cambridge, MA (URL: http://exac.broadinstitute.org) [date (03, 2015) accessed]) were also used to test for accumulation of rare variants in the coding regions of the ten TLR genes.  ExAC is a resource containing exome sequencing data from 60.706 unrelated individuals from various disease-specific and population genetic studies. The non-Finnish European population of ExAC consists of > 30.000 individuals and were used for the extraction of 237 (*TLR1*), 210 (*TLR2*), 201 (*TLR3*), 208 (*TLR4*), 213 (*TLR5*), 198 (*TLR6*), 103 (*TLR7*), 118 (*TLR8*), 369 (*TLR9*) and 195 (TLR10) polymorphisms (excluding indels) present in the coding region of the ten TLRs. For each of the 100 000 iterations, the number of expected variants were calculated using the minor allele frequency from ExAC data as the probability of finding a variant for 576 chromosomes, corresponding to the number of chromosomes in the Malmö AR patients (*TLR7* and *TLR8* were handled differently as they are located on chromosome X). The number of variants from the simulation test were then compared with the number of detected variants in the Malmö population for total number of alternative allele counts of variants with MAF <1% (one-sided tests). A score was tallied each time the simulated dataset from ExAC had an excess of variants compared to the Malmö population. The test quantity was calculated as the total score over the number of iterations.

Polymorphisms where at least one of the populations showed a MAF ≥ 0.05 were also investigated individually. First, the promoter polymorphisms were tested for Hardy-Weinberg equilibrium in the subjects with AR and in the controls. Second, differences in allele frequencies between the AR and EUR populations were tested using the normal approximation z = (p_AR_-p_EUR_)/[PQ(1/N_AR_+1/N_EUR_)]^1/2^, where p_AR_ and p_EUR_ are the observed allele frequencies in the AR and EUR populations, respectively. N_AR_ and N_EUR_ are the sample sizes of the populations and P is the weighted average of p_AR_ and p_EUR_ and Q=1-P. Thus, also in this test the results from the pools of individuals were used as proxies for individual data. As opposed to the previous tests, this test was two-sided.

**Structure analysis**

To test whether sub-populations existed between the Malmö AR patients and the 1000Genomes EUR population an analysis using Structure [6] was made. A total of 22 SNPs identified in both populations with MAF ≥ 5% located in the TLR gene regions were included in the analysis. The following parameters were used: 10 000 burn-in period, 10 000 MCMC reps after burn-in, admixture model and correlated allele frequencies. The analysis was run using *K*-values ranging from 1 to 5. Approximately equal proportions of individuals were assigned to each cluster for all values of *K*, indicating that no strong population structure exists.

**REFERENCES**

1. Li H. Tabix: Fast retrieval of sequence features from generic TAB-delimited ﬁles. *Bioinformatics* 2011; **27**:718-719.
2. Abecasis GR, Auton A, Brooks LD, DePristo MA, Durbin RM, Handsaker RE, et al. An integrated map of genetic variation from 1,092 human genomes. *Nature* 2012; **491**:56-65.
3. Danecek P, Auton A, Abecasis G, Albers CA, Banks E, DePristo MA, et al. The variant call format and VCFtools. *Bioinformatics* 2011; **27**:2156-2158.
4. Sim NL, Kumar P, Hu J, Henikoff S, Schneider G, Ng PC. SIFT web server: predicting effects of amino acid substitutions on proteins. *Nucleic Acids Res* 2012; doi: 10.1093/nar/gks539.
5. Adzhubei IA, Schmidt S, Peshkin L, Ramensky VE, Gerasimova A, Bork P, et al. A method and server for predicting damaging missense mutations. *Nat Methods* 2010; **7**:248-249.
6. Hubisz JM, Falush D, Stephens M and Pritchard JK. Inferring weak population structure with the assistance of sample group information. *Mol Ecol Resour* 2009; **9**:1322-1332.
