# Supplementary material for: Genetic variation of the Toll-like receptors in a Swedish allergic rhinitis case population
Source: BMC Med Genet. 2017 Feb 23;18:18. doi: 10.1186/s12881-017-0379-6 (PMC5322632; doi:10.1186/s12881-017-0379-6)
Supplement: Additional file 6: — Outline of the study. (DOCX 12 kb) [file 12881_2017_379_MOESM6_ESM.docx]

**Additional file 6**

Description of study design

1. Sanger sequencing of promoter regions (50 base-pairs downstream to 500 base-pairs upstream the start of exon 1) in 288 Malmö AR patients and sanger sequencing of TLR8 coding sequence
2. Ion Torrent sequencing of coding sequences (Table E6), in 288 Malmö AR patients
3. Verification and comparison of the two sequencing methods in the coding region of TLR8
4. Comparison of results found in 1-2 to the European subpopulation of the 1000Genomes project. The same base-pairs targeted by the sequencing was extracted from the database.
5. One-sided simulations and permutation tests of promoter and coding regions using 1000Genomes data
6. One-sided simulation tests for the coding sequence using data extracted from the ExAC database. These tests also used the same base-pairs as was targeted in the sequencing effort in step 1 and 2
